# Polysialic Acid Modulation of Glutamate Receptors and Synaptic Mechanisms Underlying Neuronal Plasticity

**DOI:** 10.3390/neurosci7020045

**Published:** 2026-04-15

**Authors:** Kawsar Ullah Chowdhury, Subhrajit Bhattacharya, Md Reaz Uddin, Miranda N. Reed, Soon Goo Lee, Vishnu Suppiramaniam

**Affiliations:** 1 Department of Drug Discovery and Development, Auburn University, Auburn, AL 36849, USAreedmir@auburn.edu (M.N.R.); 2 Department of Molecular and Cellular Biology, College of Science and Mathematics, Kennesaw State University, Kennesaw, GA 30144, USA; 3 Cyber-Physical Realms (CYPHR) Center, Kennesaw State University, Kennesaw, GA 30144, USA; 4 School of Pharmacy, Keck Graduate Institute, Claremont, CA 91711, USA

**Keywords:** neural cell adhesion molecules, polysialic acid, glutamate, AMPA, NMDA, memory, LTP, LTD

## Abstract

Polysialic acid (PSA), a highly negatively charged glycan attached mainly to the neural cell adhesion molecule (NCAM), is emerging as a critical but underrecognized extracellular regulator of glutamatergic neurotransmission. While previous literature has focused on PSA’s developmental roles, increasing evidence indicates that PSA–NCAM also contributes to synaptic plasticity mechanisms in the mature brain. This review integrates evidence from structural biophysics, single-channel electrophysiology, and disease models to explain how PSA modulates glutamate receptor gating to control learning and memory. We synthesize findings from biochemical reconstitution, electrophysiological recordings, and in vivo studies to show that PSA can modulate α-amino-3-hydroxy-5-methyl-4-isoxazolepropionic acid (AMPA) receptor open probability, burst duration, and cooperative gating without affecting conductance, thereby promoting long-term potentiation. Conversely, PSA selectively suppresses GluN2B-containing extrasynaptic N-methyl D-Aspartate (NMDA) receptor activity by lowering open probability and calcium influx, maintaining an optimal balance between potentiation and depression while providing neuroprotection. Disruption of PSA–NCAM signaling in developmental and disease models, including prenatal cannabinoid exposure and neurodegeneration, produces cognitive deficits reversible by PSA restoration. Notably, much of the current evidence derives from in vitro systems, with relatively few studies conducted in vivo, and studies employing PSA mimetics mostly, which should be considered when interpreting physiological relevance. Collectively, the available evidence suggests that PSA functions as an extracellular modulator linking synaptic glycans to glutamate receptor regulation and plasticity related signaling pathways, highlighting the potential importance of extracellular glycan mechanisms in the control of synaptic function.

## 1. Introduction

Synaptic plasticity is a cellular mechanism underlying learning and memory, characterized by activity-dependent changes in synapses within the mammalian brain [1]. Synaptic plasticity can be controlled by coordinated actions of both excitatory and inhibitory neurotransmission [2]. In excitatory synapses, learning and memory are primarily regulated by glutamate, the brain’s major excitatory neurotransmitter [3]. Coordinated signaling through excitatory and inhibitory receptors determines synaptic strengthening and scaling in the brain, which eventually modulates learning and memory [3,4,5]. The most important of these excitatory receptors are α-amino-3-hydroxy-5-methyl-4-isoxazolepropionic acid (AMPA) and N-methyl D-Aspartate (NMDA) type glutamate receptors, which, due to their inherent subunit diversity, endow synaptic transmission with graded control via a slow calcium-mediated inward current [6]. While previous research has primarily focused on intracellular signaling cascades [7], receptor phosphorylation [8], and trafficking mechanisms [9], it has become increasingly clear that the extracellular synaptic environment plays an equally critical role in shaping glutamate receptor function and plasticity outcomes [10,11].

Among extracellular modulators, glycans have emerged as potent regulators of synaptic organization and signaling [11,12,13]. In particular, several articles have been published on polysialic acid (PSA), a significant, highly negatively charged glycan, due to its role in regulating neuronal development, synaptic remodeling, and plasticity [14,15]. PSA profoundly modifies the function of neural cell adhesion molecule (PSA–NCAM) post-translationally [14,15], subsequently acting as a dynamic regulator of synaptic structure and function. The roles of the extracellular matrix (ECM), glycans, and PSA–NCAM in neural development and synaptic plasticity have been extensively reviewed previously [12,13,14,15]. These studies established that extracellular glycoconjugates contribute to synapse formation, structural remodeling, and activity-dependent circuit refinement. However, these studies have primarily focused on structural and developmental roles, with less emphasis on how extracellular components directly regulate synaptic signaling at the level of the glutamate receptor function.

Through these interactions, PSA can modulate the function of AMPA and NMDA subtypes of glutamate receptors. However, most existing reviews primarily focus on developmental neurobiology, ECM signaling, or cell adhesion mechanisms rather than on the biophysical regulation of glutamate receptor activity at the level of ion-channel gating and synaptic signaling dynamics. In this review, we therefore summarize current knowledge of the structural and biophysical properties of PSA and examine how PSA–NCAM interactions modulate the function of AMPA and NMDA receptors (NMDAR) at the molecular, synaptic, and circuit levels. We focus particularly on insights gained from single-channel electrophysiology, biochemical reconstitution, and in vivo models of synaptic plasticity and cognitive dysfunction [16]. To identify relevant articles for this review, a comprehensive literature search was conducted in PubMed and Google Scholar using the following keywords: “polysialic acid”, “polysia”, “PSA”, “PSA mimetic”, “Neural Cell Adhesion Molecule”, “PSA–NCAM”, “NCAM polysialylation” and its effect on “single-channel”, “single-channel electrophysiology”, “glutamate receptors”, “AMPA receptors”, “synaptic NMDA receptors”, “extrasynaptic NMDA receptors”, and terms related to the specific effect discussed in this review. This review includes and synthesizes literature published up to March 2026. Studies were selected based on relevance to PSA–NCAM biology, glutamate receptor modulation, and synaptic plasticity mechanisms. Priority was given to primary experimental studies providing mechanistic insight into receptor electrophysiology, synaptic physiology, or structural properties of polysialic acid. Additional references describing extracellular matrix signaling, receptor organization, and disease models were included when they provided important conceptual context for PSA-dependent regulation of glutamatergic signaling. As this manuscript is a narrative review, we prioritized primary mechanistic studies on single-channel electrophysiology and neuronal electrophysiology, with a focus on perturbations of PSA in neurons or brain slices, supplemented by selected in vivo and disease-model studies.

## 2. Structure of Polysialic Acid

**Figure 1:**
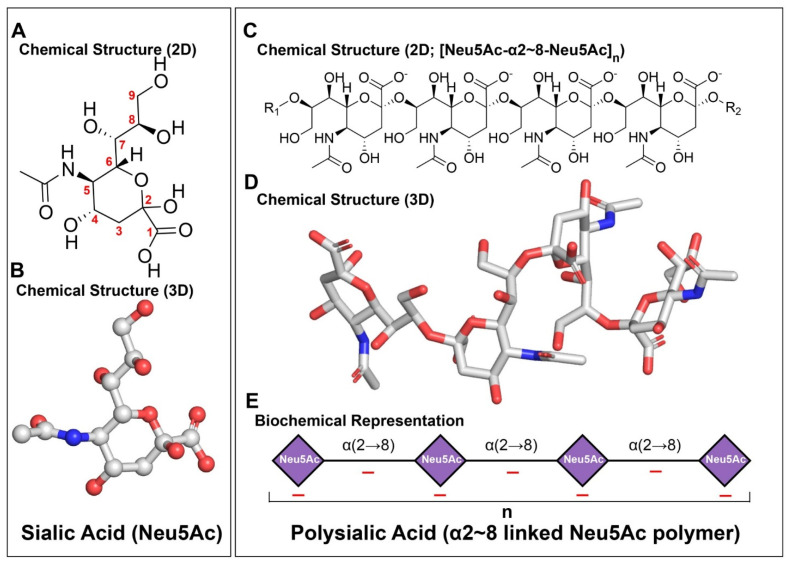
Structures of sialic acid (Neu5Ac) and polysialic acid (PSA). (**A**,**B**) Representative 2D and 3D chemical structures of Neu5Ac. (**C**,**D**) Representative 2D and 3D chemical structures of PSA, illustrating the α(2→8)-linked Neu5Ac repeat. (**E**) Schematic representation of PSA showing the repeating Neu5Ac units and polymer length (n).

Polysialic acid is a long, linear homopolymer of sialic acid that has a profound effect on neuronal functions [12]. To date, more than 50 structurally distinct forms of sialic acid have been identified across biological systems [17]. However, only a limited subset of these sialic acids participates in the formation of PSA, primarily N-acetylneuraminic acid (Neu5Ac), N-glycolylneuraminic acid (Neu5Gc), and deaminoneuraminic acid (Kdn) (Figure 1A,B) [17]. The structural diversity of sialic acids arises from substitutions of one or more hydroxyl groups at the monomeric level, such as acetylation, methylation, phosphorylation, or sulfation [17]. In contrast, PSA in mammals is composed exclusively of Neu5Ac residues, forming a chemically uniform homopolymeric structure (Figure 1C,D) [18]. PSA is expressed both pre- and post-synaptically [19].

In mammalian PSA, individual Neu5Ac residues are predominantly linked through α2,8 glycosidic bonds formed between the anomeric C2 carbon of one residue and the C8 hydroxyl group of the adjacent residue (Figure 1A–D). This linkage helps in the formation of a long chain of sialic acid, ranging from approximately 8 to over 400 residues, depending on the cellular context and developmental stage (Figure 1E) [20]. The α2,8 linkage geometry confers substantial rotational freedom between adjacent sialic acid residues, which ultimately facilitates the formation of a highly flexible, extendable, and dynamically fluctuating polymer in solution [21,22].

In the nervous system, PSA is most prominently expressed as a post-translational modification of the neural cell adhesion molecule (NCAM). PSA chains are covalently attached via O-glycosidic linkages to N-linked glycans located primarily within the fifth immunoglobulin-like domain of NCAM [15]. The polysialylation of NCAM by PSA extends the NCAM structure by adding negatively charged, long, flexible polysialic acid chains to its surface. This modification disrupts homophilic NCAM-NCAM interactions through steric and electrostatic effects. As a result, NCAM functions as a versatile regulator of cell–cell communication and intracellular signaling [23].

Besides modulating adhesion, PSA also influences the local physicochemical environment surrounding membrane proteins. Though it has a net negative charge, PSA can still bind divalent cations such as Ca^2+^ and Mg^2+^ via coordination with carboxylate groups, which can eventually alter local ionic distributions at the cell surface [24]. Through its ion-binding capacity and flexible conformation, PSA functions as an electrostatic buffer. This way, PSA modulates charge screening and establishes ionic microdomains near the membrane. In addition, PSA interacts with a range of extracellular proteins, including growth factors and guidance molecules, through mechanisms that depend on polymer length and flexibility rather than discrete binding motifs [25,26]. Such interactions highlight the importance of PSA’s polymeric nature, as its functional influence arises from collective physicochemical properties rather than sequence-specific recognition.

Importantly, PSA exists in multiple length distributions in vivo [27,28]. Different chain lengths have different effects. Longer chains produce larger hydrated exclusion volumes and increase intermembrane spacing [29], whereas shorter fragments or soluble PSA may behave as extracellular modulators [30]. In addition, membrane-associated poly/oligoSia can alter surface charge density and local electrostatic microenvironments [31].

## 3. Glutamate Receptors and Their Subunits

The glutamatergic system is the principal mediator of excitatory synaptic transmission in the mammalian central nervous system [3,6]. Glutamate can bind with both metabotropic and ionotropic glutamate receptors [6,32]. Among the ionotropic receptors, AMPA receptor (AMPAR) subtype of glutamate receptors modulates fast excitatory synaptic transmission, whereas activation of the NMDA subtypes of glutamate receptors produces slower, long-lasting synaptic responses due to their voltage dependence and slower channel kinetics [33,34]. AMPARs have four major subunits, GluA1-GluA4, that assemble to form the tetrameric glutamate receptors [6,34]. On the other hand, NMDARs are heterotetrameric in type, composed of two obligatory GluN1 subunits in combination with two regulatory GluN2 (GluN2A-GluN2D), which may form di- or triheteromeric assemblies (two different GluN2 combinations) and/or the pH sensitive GluN3 (GluN3A-GluN3B) subunits [33,35,36]. Region specific localization, spatio-temporal expression, and diverse interaction with other neurotransmitter systems, combined with physical interaction with NCAMs bestow these receptors unprecedented leverage over synaptic plasticity, making them critical therapeutic targets in several disease states related to dementia and memory loss [37,38,39].

PSA is therefore well positioned to modulate ionotropic glutamate receptors because it can reshape the synaptic extracellular microenvironment through two complementary mechanisms: (i) electrostatic buffering and ion-binding that can alter local charge screening near the membrane [24,31], and (ii) steric, hydrated exclusion effects that increase intermembrane spacing and reduce adhesion-mediated constraints on receptor mobility [23,29]. Together, these PSA-dependent changes can influence the nanoscale clustering and lateral diffusion of AMPARs/NMDARs and thereby bias channel gating behavior and synaptic efficacy without requiring a classical ligand–receptor interaction [11,13,37].

## 4. PSA Modulation of Glutamate Receptors and Synaptic Plasticity

PSA does not modulate glutamate receptor function through the classic ligand–receptor binding mechanism. Instead, PSA modulates AMPA-type glutamate receptor function by changing the biophysical properties and nanoscale organization of the synapse, a process known as homeostatic synaptic scaling, a mechanism of plasticity that grades strengths of excitatory synapses to stabilize activity [40]. Through these modulations of glutamatergic signaling, PSA controls long-term potentiation (LTP) and long-term depression (LTD), the key cellular processes underlying learning and memory [41,42,43].

Most studies on PSA’s impact on glutamate receptors use colominic acid, a bacterial PSA mimetic [40,41]. Colominic acid is a homopolymeric α(2→8)-linked polysialic acid derived from bacterial capsules [44] and is commonly used experimentally to mimic the physicochemical properties of mammalian PSA [41]. However, it should be noted that colominic acid represents a soluble polymer that lacks the native membrane anchoring and spatial organization provided by NCAM in mammalian neurons. Therefore, while colominic acid reproduces important electrostatic and polymeric features of PSA, it does not fully recapitulate the structural and signaling context of endogenous PSA–NCAM complexes present at synapses.

Investigation of single-channel electrophysiological activity of receptors is an important method to determine neuronal cell activity, because any change in single-channel activity of receptors can alter the overall synaptic currents that control LTP and LTD [45]. While recording single channel activity, the commonly measured parameters are probability of opening of an ion-channel (the fraction of time the channel spends in the open state) [40,41], unitary conductance (the amplitude of single channel currents that reflects the flow of ion) [46] and burst activity (clusters of repeated openings separated by brief closures) [47].

### 4.1. Effect of PSA on AMPA Receptor Activity

Extensive biochemical and electrophysiological research in previously published articles has shown that PSA does not directly bind to the agonist-binding or transmembrane domains of AMPARs; instead, its effects are mediated primarily through PSA–NCAM interactions that influence the lateral mobility, clustering, and gating behavior of synaptic AMPARs [48,49].

The highly negatively charged and hydrated PSA chain creates a steric and electrostatic barrier within the synaptic cleft, reducing the adhesive constraints imposed by NCAM–NCAM or NCAM-extracellular matrix interactions and thereby increasing the potential for synaptic plasticity [12,14,15]. When investigated at the single-channel level using artificially reconstituted lipid bilayers and synaptosomes, PSA has been found to enhance the single-channel open probability of AMPARs [40]. Notably, PSA increased the burst duration and the mean open-time of AMPAR-mediated single-channel activity, while reducing the interburst interval and the mean closed-time. In the acutely isolated juvenile hippocampal neurons, PSA potentiated AMPAR-mediated currents. PSA has also been found to modulate the activity of drugs that act on AMPAR [50]. In the absence of PSA, the drugs were ineffective on AMPARs, leading to altered activity.

**Figure 2:**
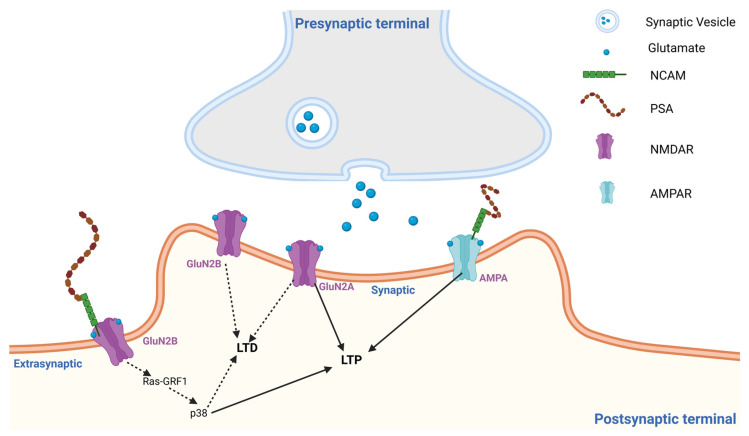
Schematic model of polysialic acid (PSA) modulation of glutamate receptor signaling. PSA attached to NCAM alters the extracellular synaptic environment and receptor organization. PSA has been reported to enhance AMPAR-mediated synaptic activity that promotes LTP while selectively suppressing extrasynaptic GluN2B-containing NMDAR signaling associated with Ras-GRF1/p38 pathways and LTD. Created in BioRender. The figure was created with Biorender.com.

From a biophysical perspective, PSA shares functional similarities with other polyanionic glycans such as heparan sulfate [51,52], dextran sulphate, and fucoidan [53] in that its highly hydrated and negatively charged polymeric structure can potentially lower the energy barrier for receptor conformational transitions and cooperative channel gating without engaging classical binding pockets. Overall, the articles referenced above demonstrate that PSA–NCAM, due to its long, charged, polymeric structure, boosts AMPAR function. As illustrated in Figure 2, PSA–NCAM achieves this by promoting AMPAR-mediated synaptic signaling, by enhancing receptor mobility and clustering at the postsynaptic density, which is associated with increased channel open probability and burst activity.

In addition to these kinetic effects, PSA-dependent receptor organization may also influence the cooperative behavior of AMPA receptor channels. In our previous publications, we have shown that AMPA receptors show a cooperative channel-gating property [54]. Cooperative opening of the channels represents the non-independent, synchronous opening among nearby glutamate receptor channels. PSA plays a role in facilitating such cooperative gating among synaptic glutamate receptors by promoting the lateral mobility and clustering of AMPARs within nanoscale regions of the postsynaptic density [30,40,48]. This organization of receptors can facilitate coordinated and synchronized openings of neighboring AMPAR channels during synaptic activation. Such coordinated channel activity can amplify postsynaptic responses to a given glutamate transient by increasing channel open probability and burst activity without altering single-channel conductance. Therefore, cooperative gating can be viewed as a functional consequence of PSA-mediated receptor mobility and clustering rather than as a completely independent phenomenon.

Degradation or loss of PSA disrupts this receptor organization, which may lead to disorganized receptor gating and reduced synaptic efficiency. Through these mechanisms, PSA contributes to strengthening synaptic responses without altering channel conductance, thereby supporting synaptic plasticity mechanisms involved in learning and memory. Taken together, these findings are largely derived from studies using PSA mimetics and reduced experimental systems, and while the observed effects on channel kinetics are well supported, the underlying mechanistic interpretations remain hypothetical and context-dependent.

### 4.2. Effect of PSA on NMDA Receptor Activity

While modulating NMDAR function, PSA acts as a regulator between synaptic NMDAR activity, which supports synaptic plasticity and neuronal survival, and extrasynaptic NMDAR signaling, which is linked to LTD, cellular stress, and degeneration [30]. PSA alters the extracellular and postsynaptic signaling environment, rather than the typical ligand–receptor binding [14]. The effect of PSA–NCAM is selective. It reduces the activity of extrasynaptic GluN2B-containing NMDARs by decreasing the probability of opening and calcium influx through NMDAR (Figure 2), without altering single-channel conductance [48]. Biophysically, an unchanged unitary conductance indicates that PSA does not alter the permeation properties of the NMDAR channel. Instead, the reduced Ca^2+^ entry is best explained by possible gating-level modulation. In single-channel terms, PSA lowers Ca^2+^ influx primarily by reducing open probability through changes in channel kinetics (i.e., fewer/shorter openings and/or longer closures that reduce burst activity), rather than by changing the single-channel current amplitude or by a membrane potential–dependent mechanism. Yet the synaptic NMDAR-dependent activity is preserved, which eventually helps in LTP. In fact, after PSA removal, extrasynaptic GluN2B-mediated Ca^2+^ transients were increased, indicating a gating level modulation rather than pore blockade or subunit removal of NMDAR. Importantly, an increase in Ca^2+^ influx following PSA removal should not be interpreted as inherently pathological. Moderate and spatially restricted Ca^2+^ transients through synaptic NMDARs are well established as essential triggers for physiological synaptic plasticity, including LTP induction. However, excessive or prolonged Ca^2+^ entry, particularly through extrasynaptic GluN2B-containing NMDARs, preferentially activates signaling pathways associated with LTD, cellular stress responses, and excitotoxic mechanisms (Figure 2). In this context, PSA appears to function as a regulatory buffer that constrains excessive extrasynaptic Ca^2+^ signaling while preserving the physiological Ca^2+^ dynamics required for synaptic plasticity and learning. Electrophysiological recordings from hippocampal neurons demonstrate that the enzymatic removal of PSA using endoneuraminidase-N (endo-NF) significantly enhances NMDA-evoked currents and increases extrasynaptic GluN1/GluN2B mediated synaptic transmission [55]. At the same time, exogenous PSA or intact PSA–NCAM reduces NMDAR activity, an effect that is occluded by the GluN2B-selective antagonist ifenprodil, directly implicating GluN2B-containing receptors as the primary target [30].

PSA regulates this extrasynaptic GluN2B-mediated signaling by controlling the influx of excessive calcium and preventing the overactivation of calcium-dependent pathways, such as Ras-GRF1, p38 MAPK, and other stress-associated cascades, which can alter synaptic plasticity when hyperactive [48]. Importantly, the subunit composition of NMDARs differ between synaptic and extrasynaptic compartments. The synaptic NMDARs are predominantly GluN1/GluN2A/GluN2B triheteromeric (2A/2B) assemblies that help in the physiological influx of Ca^2+^, necessary for LTP. In contrast, extrasynaptic receptors are enriched in GluN1/GluN2B diheteromeric (2B-only) assemblies that allow increased amounts of calcium influx associated with LTD. When tested for single-channel activity of GluN1/2A/2B and GluN1/2B-containing NMDA channels, PSA was found to decrease open probability without altering conductance [48], thereby selectively controlling the calcium entry through extrasynaptic GluN2B receptors while preserving normal synaptic receptor function. However, PSA had no effect on GluN1/2A-containing NMDAR channels. The learning and memory deficit in these PSA-deficient mice is rescued by pharmacological GluN2B receptor blockade. In fact, in PSA deficient mice, a short fragment of PSA named NANA-12 rescued the impaired performance when investigated for mPFC dependent cognitive tasks [55]. In a similar study, PSA was found to inhibit NMDAR currents in a concentration-dependent manner, reducing their open probability [30]. These findings indicate a strong correlation between PSA and NMDAR-mediated signaling, as well as synaptic plasticity. PSA also protects against glutamate-induced excitotoxicity, further highlighting its role in balancing calcium signaling and controlling synaptic plasticity [30]. Quantitative comparisons across studies remain limited due to variability in experimental systems and reporting approaches. In a separate study using a rat model of prenatal cannabinoid exposure (PCE), researchers observed that PCE-induced memory impairments were associated with reduced PSA–NCAM expression [41]. The selective suppression of extrasynaptic GluN2B signaling pathways is illustrated schematically in Figure 2.

### 4.3. Effect of PSA on Glutamate Receptor-Mediated Synaptic Plasticity, Learning, and Memory

Synaptic plasticity is the cellular basis for learning and memory. At the functional level, enzymatic degradation of PSA by endo-NF consistently reduces LTP in hippocampal CA1 synapses [56,57], an effect that can be attributed to both impaired AMPAR recruitment and stabilization during activity-dependent synaptic strengthening [58] as well as the impairment of NMDAR expression and/or activity [39].

Previous studies have shown that in PCE animals, NMDAR activity is increased due to decreased PSA levels [41]. This reduction increases the open probability of NMDARs at the single-channel level, leading to increased LTD and decreased LTP. This alteration in synaptic plasticity indicates enhanced calcium influx and a heightened susceptibility to Ca^2^-dependent excitotoxicity following PCE. When treated with the PSA mimetic colominic acid, PCE rats showed enhanced LTP and decreased LTD, further underscoring the neuroprotective role of PSA. PSA has been found to be involved in the induction of LTP also [59]. Application of PSA increased LTP in NCAM-deficient mice, indicating that PSA is the main component that facilitates both the induction and the increase in LTP [59]. As illustrated in Figure 2, PSA–NCAM modifies the extracellular synaptic environment in a manner that enhances AMPAR-mediated synaptic transmission while selectively restricting extrasynaptic GluN2B-NMDAR signaling, thereby influencing the balance between LTP and LTD-associated signaling pathways.

Overall, PSA modulates both the structure and function of both AMPARs and NMDARs, linking PSA to synaptic plasticity and memory through the regulation of signaling dynamics rather than traditional ligand–receptor mechanisms [37]. The effect of PSA on glutamate receptors and, eventually on synaptic plasticity is summarized in Table 1. While the above sections describe the mechanistic effects of PSA on glutamate receptor function, these findings collectively point to broader implications for synaptic regulation, disease mechanisms, and therapeutic potential, which are discussed below.

### 4.4. Integrated Implications of PSA-Mediated Glutamatergic Regulation

Taken together, the mechanistic findings described above highlight the role played by PSA as a key extracellular component of the synapse, controlling glutamate-mediated neurotransmission. This control is made possible by both the biophysical and structural modulation of glutamate receptors. Rather than functioning solely through classical ligand–receptor interactions, PSA represents an extracellular regulatory layer that influences receptor organization, gating behavior, and synaptic signaling through its physicochemical properties within the synaptic microenvironment. These observations further emphasize the importance of the synaptic extracellular milieu as an active regulator of receptor behavior and plasticity outcomes [11,61].

Although this review focuses on PSA–NCAM, other extracellular matrix components also regulate glutamatergic synapses. For example, astrocyte-derived glypicans 4 and 6, which are heparan sulfate proteoglycans (HSPGs), promote excitatory synapse maturation by increasing the surface clustering and synaptic recruitment of AMPARs. Thus, HSPGs and PSA–NCAM likely represent complementary extracellular regulators of glutamatergic function [62].

Single-channel electrophysiological analyses and synaptosomal reconstitution experiments demonstrate that PSA selectively enhances the open probability of AMPARs, burst duration, and cooperative gating, while leaving unitary conductance unchanged [40,41].

In contrast to its facilitatory effects on AMPARs, PSA exerts a constraining influence on NMDAR signaling, particularly on GluN2B-containing receptors. Its effect on GluN2B-containing NMDARs is selective, as previous studies have shown that PSA suppresses GluN2B-mediated NMDAR activity by reducing the channel’s open probability and calcium influx, without altering single-channel conductance [30,48]. Thus PSA helps in maintaining the NMDAR signaling within an optimal dynamic range, confers neuroprotection, and preserves synaptic plasticity.

Removing or degrading PSA has been found to disrupt the balance between LTP and LTD, leading to impaired spatial and contextual memory [42,48,57]. The PSA theory was further supported when this memory deficit was rescued by the pharmacological inhibition of GluN2B-containing NMDARs [48]. Such findings reinforce the concept that PSA does not simply enhance excitatory transmission but instead fine-tunes synaptic responsiveness to support stable learning.

Beyond physiological plasticity, alterations in PSA expression have important implications for disease and developmental perturbations. Lower levels of PSA–NCAM have been found to increase NMDAR activity, disrupt synaptic plasticity, and cognitive impairments in models of prenatal cannabinoid exposure, neurodevelopmental disorders, and neurodegenerative conditions [37,56]. In these contexts, restoration of PSA levels rescues synaptic and behavioral deficits, highlighting PSA as a potential therapeutic target for conditions characterized by dysregulated glutamatergic signaling and calcium homeostasis. Evidence from human clinical and neuropathological studies further supports the idea that alterations in PSA–NCAM signaling are associated with several neuropsychiatric and neurodegenerative conditions, highlighting the broader relevance of extracellular glycan regulation in synaptic plasticity. Genetic investigations have identified variants in enzymes responsible for polysialic acid biosynthesis, as susceptibility factors for psychiatric illnesses such as schizophrenia and bipolar disorder, suggesting that disruption of PSA dependent neuronal remodeling pathways may contribute to disease vulnerability [63]. Consistent with this concept, clinical studies have reported elevated circulating PSA levels in individuals with schizophrenia spectrum disorders, and these increases were associated with structural reductions in hippocampal gray matter, an area critically involved in glutamatergic synaptic plasticity and memory formation [64]. Additional neuroimaging work has shown that altered PSA–NCAM levels correlate with structural changes in the prefrontal cortex and cognitive impairments in schizophrenia, further implicating dysregulated polysialylation in cortical circuit abnormalities linked to higher cognitive function [65]. Post-mortem analyses of the dorsolateral prefrontal cortex have also revealed reduced PSA–NCAM expression in schizophrenia, accompanied by alterations in synaptic markers associated with inhibitory and excitatory neurotransmission, indicating that PSA-dependent structural plasticity may influence the balance of cortical network activity [66]. In neurodegenerative conditions, decreased PSA–NCAM immunoreactivity has been observed in the entorhinal cortex of Alzheimer’s disease (AD) brains and was inversely related to tau pathology, supporting the idea that impaired PSA-mediated synaptic remodeling may contribute to progressive synaptic dysfunction during disease progression [67]. A recent study in AD showed increased extrasynaptic GluN2B localization together with reduced PSA–NCAM expression, particularly in the hippocampus and cortex, supporting a shift toward pathological glutamatergic signaling during disease progression [16]. Although genetic linkage analyses have not consistently implicated the NCAM locus itself in schizophrenia susceptibility, these converging findings suggest that the dysregulation of PSA-dependent extracellular mechanisms may alter glutamatergic synaptic signaling, including AMPA receptor activity and NMDA receptor-dependent plasticity, thereby contributing to cognitive deficits observed in both psychiatric and neurodegenerative disorders [68].

Beyond plasticity, PSA-mediated modulation of NMDARs confers protection against glutamate-induced excitotoxicity. The PSA functions as a regulator in the synaptic environment by limiting excessive calcium entry, thereby controlling downstream signaling pathways, including p38 MAPK and Ras-GRF1 [30,48]. This protective mechanism puts PSA as a future potential therapeutic strategy to preserve synaptic plasticity while minimizing neurotoxicity, an advantage over global NMDAR antagonists.

PSA dysregulation has also been implicated in neurodevelopmental insults such as prenatal cannabinoid exposure. Previously, we have reviewed the effect of cannabis on glutamate-mediated neurotransmission [69]. Cannabis exposure has notable effects on PSA–NCAM expression, which eventually influences learning and memory processes. Experimental models of prenatal cannabis exposure decrease PSA–NCAM expression that eventually causes learning and memory deficits. This deficit is accompanied by increased NMDAR-mediated single-channel activity. Earlier, we discussed that PSA decreases calcium entry and provides neuroprotection. In the PCE rats, treatment with PSA also increased LTP, possibly by decreasing calcium entry and reducing NMDAR single-channel activity [41].

Despite substantial progress in defining the role of PSA in glutamatergic synaptic plasticity, several important limitations remain. First, much of the mechanistic insight into PSA-dependent modulation of AMPARs and NMDARs derives from studies using bacterially derived PSA (e.g., colominic acid). Although colominic acid reproduces the polymeric and electrostatic characteristics of PSA, it does not replicate the precise molecular context of PSA attached to NCAM within the neuronal membrane. In vivo, PSA is presented as a post-translational modification of NCAM that influences cell adhesion, receptor organization, and synaptic architecture. Therefore, while colominic acid experiments provide important insight into the physicochemical effects of PSA polymers on glutamate receptor activity, they should be interpreted together with studies manipulating endogenous PSA–NCAM expression to fully understand physiological mechanisms. But complementary approaches such as the enzymatic removal of PSA by endo-N or genetic manipulation of polysialyltransferases provide additional experimental support for the physiological role of PSA in regulating glutamatergic signaling. While these approaches provide critical biophysical resolution, they may not fully recapitulate the spatial constraints, molecular crowding, and dynamic regulation of PSA–NCAM interactions present in intact synapses.

**Figure 3:**
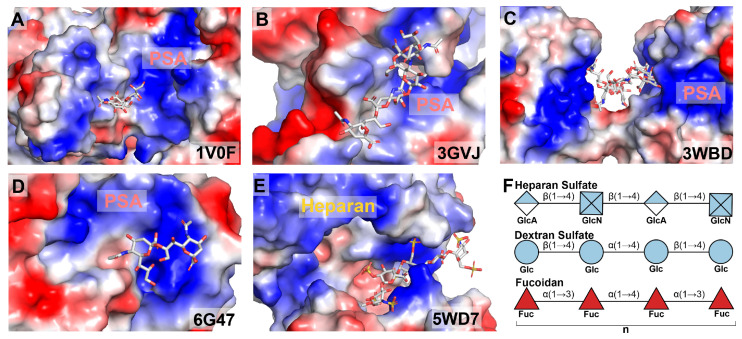
Electrostatic surface representations of PSA-binding proteins. (**A**–**E**) Negatively charged polysialic acid (PSA) and its analogs bind to positively charged binding pockets, including the polysialic acid-degrading endosialidase from *Escherichia* phage K1F (panel (**A**); PDB ID: 1V0F) [74], endo-N-acetylneuraminidase from *Escherichia* phage K1F (panel (**B**); PDB ID: 3GVJ) [73], an anti-polysialic acid antibody single-chain Fv fragment from *Mus musculus* (panel (**C**); PDB ID: 3WBD) [76], the short fiber knob from human adenovirus 52 (panel (**D**); PDB ID: 6G47) [75], and a bacterial polysialyltransferase from *Mannheimia haemolytica* (panel (**E**); PDB ID: 5WD7) [77]. Electropositive regions are shown in blue, and electronegative regions are shown in red. The corresponding PDB ID is indicated in each panel. (**F**) Schematic representation of representative backbone structures of other polyanionic glycans. Glc, glucose; GlcN, glucosamine; GlcA, glucuronic acid; Fuc, fucose.

Second, the atomic-level conformations of PSA, the determinants of binding specificity, and the molecular basis of PSA-binding partner interactions remain poorly understood. This knowledge gap hinders a comprehensive understanding of how PSA interacts with its diverse partners in cells. Multiple nuclear magnetic resonance (NMR) studies combined with molecular modeling and molecular dynamics (MD) simulations have suggested flexible helical conformations for PSA; however, these proposed models lack consistency [22,70,71,72]. Only a few structures featuring polymeric PSA ligands are available in the Protein Data Bank (PDB) (Figure 3B–E). Although only a few structures featuring polymeric PSA ligands are available in the Protein Data Bank (PDB), structural and computational studies consistently demonstrate that PSA-binding proteins typically present prominent electropositive clefts or surface patches that accommodate the polyanionic PSA chain through complementary electrostatic interactions. While the details of recognition vary among proteins for PSA and structurally related anionic oligosaccharides (e.g., fondaparinux) (Figure 3F), this electropositive binding mechanism is a conserved feature [73,74,75,76,77]. Supporting these structural models, site-directed mutagenesis coupled with in vitro and in vivo binding assays demonstrates that the substitution of key basic residues results in complete or significant loss of PSA binding, underscoring the critical role of electrostatic interactions in PSA recognition [75,76]. These complementary analyses reveal the molecular mechanisms underlying PSA recognition by AMPARs and NMDARs.

Third, while PSA-mediated suppression of GluN2B-containing NMDARs has been well documented, it remains unclear how PSA dynamically regulates subunit switching during development and aging, particularly during the transition from GluN2B- to GluN2A-dominated synapses [33]. Addressing this question will be crucial for understanding how extracellular glycans influence plasticity throughout the lifespan. In this context, it is notable that extracellular matrix signaling pathways beyond PSA–NCAM can also shape NMDAR subunit composition and plasticity. Reelin signaling through the apolipoprotein E receptor 2 (ApoER2) has been shown to regulate the maturational decline in GluN1/GluN2B contribution and promote features consistent with the developmental GluN2B-to-GluN2A shift [78]. ApoER2 is part of a postsynaptic complex with NMDARs, and Reelin–ApoER2 signaling enhances synaptic plasticity in part through tyrosine phosphorylation and the functional modulation of NMDAR subunits [79]. Together, these studies suggest that PSA-dependent regulation of GluN2B signaling may intersect with—or be complemented by—Reelin–ApoER2 pathways that influence NMDAR maturation and synaptic plasticity, which should be explored in future work.

Future work should also explore how PSA interacts with other components of the synaptic glycan, including heparan sulfate proteoglycans and gangliosides, which are increasingly recognized as modulators of ion channel function and synaptic stability [12,13]. Integrating glycan biology with computational modeling of synaptic electrostatics may provide new insights into how extracellular charge landscapes regulate neuronal computation.

## 5. Conclusions

**Figure 4:**
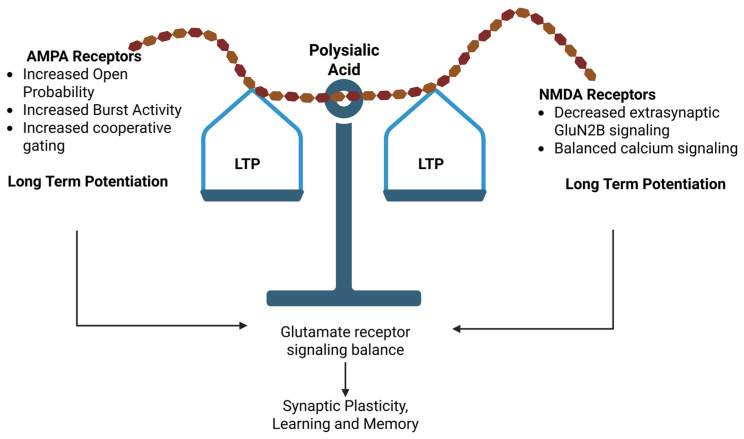
Polysialic acid-mediated regulation of synaptic plasticity. Polysialic acid (PSA) regulates the balance between long-term potentiation (LTP) and long-term depression (LTD) by influencing AMPA and NMDA receptor function and signaling at the synapse, thereby modulating synaptic plasticity, learning and memory. Created in BioRender. The figure was created with Biorender.com.

In summary, the studies discussed in this review support the role of PSA as an extracellular regulator of glutamatergic synaptic signaling. Through its association with NCAM and its highly charged, hydrated polymeric structure, PSA can influence receptor mobility, channel gating behavior, and the balance between AMPAR- and NMDAR-dependent signaling pathways that contribute to synaptic plasticity. As illustrated schematically in Figure 4, PSA-mediated modulation of glutamate receptor activity can influence the relative balance between LTP and LTD, thereby affecting synaptic mechanisms associated with learning and memory.

However, current evidence primarily derives from biochemical, electrophysiological, and experimental model systems, and the precise in vivo specificity and physiological scope of PSA-dependent mechanisms remain to be fully established. Future studies integrating high-resolution imaging, single-molecule electrophysiology, and circuit-level analyses will therefore be essential to clarify how extracellular glycan mechanisms such as PSA contribute to synaptic regulation in both normal brain function and neurological disease.

## Figures and Tables

**Table 1:** Summary of the effects of PSA on AMPA and NMDA receptor activity.

Properties	Effect	Source
AMPAR open probability	Increased by colominic acid	[40]
Burst duration of AMPAR	Increased by colominic acid	[40]
Mean open time in bursts within bursts of AMPAR	Increased by colominic acid	[40]
Interburst interval of AMPAR	Decreased by colominic acid	[40]
Mean close time of AMPAR	Decreased by colominic acid	[40]
AMPAR currents	Potentiated by colominic acid	[40]
GluN1/2A/2B current	Decreased open probability but no change in conductance	[48]
GluN1/2B current	Decreased open probability but no change in conductance	[48]
GluN1/2B current	Increased after PSA removal	[55]
GluN1/2A current	No significant effect	[48]
GluN2B mediated Ca^2+^ transients	Increased after acute removal of PSA	[48]
LTP	Impaired in PSA deficient mice	[48]
NMDAR current	Inhibited by PSA	[30]
GluN2B containing NMDAR current	Inhibited by PSA	[30]
Open probability of GluN2B-containing NMDAR	Decreased (concentration dependent) by PSA	[30]
GluN2B-lacking NMDAR	No effect	[30]
NMDAR-mediated excitotoxicity	Inhibited by PSA	[30]
LTP rescue in PCE mice	Restored by PSA	[41]
Basal synaptic transmission	Decrease in PSA deficient mice	[60]
LTP	Increased with PSA	[59]
Impaired cognition in PSA deficient mice	Rescued by short fragment of PSA (e.g., NANA-12)	[55]

## Data Availability

No new data were created or analyzed in this study. Data sharing is not applicable to this article.

## Abbreviations

The following abbreviations are used in this manuscript:

AD	Alzheimer’s disease
AMPA	α-amino-3-hydroxy-5-methyl-4-isoxazolepropionic acid
AMPAR	α-amino-3-hydroxy-5-methyl-4-isoxazolepropionic acid receptor
ECM	Extracellular Matrix
LTP	Long term potentiation
LTD	Long term depression
NCAM	Neural Cell Adhesion Molecule
Neu5Ac	N-acetylneuraminic acid
Neu5Gc	N-glycolylneuraminic acid
NMDA	N-methyl D-aspartate
NMDAR	N-methyl D-aspartate receptor
PCE	Prenatal cannabinoid exposure
PSA	Polysialic Acid
